# A descriptive study of orthopaedic injuries due to parachute jumping in soldiers

**DOI:** 10.1186/s12873-020-00354-7

**Published:** 2020-07-31

**Authors:** Taner Sahin, Sabri Batın

**Affiliations:** 1 Emergency Medicine Clinic Kayseri City Education and Research Hospital affiliated with University of Health Sciences Turkey, 38090 Kayseri, Turkey; 2 Orthopedist, Orthopedic Clinic Kayseri City Education and Research Hospital affiliated with University of Health Sciences Turkey, 38090 Kayseri, Turkey

**Keywords:** Parachuting injuries, Abbreviated injury scale, Injury severity score

## Abstract

**Background:**

While parachute jumping, soldiers may suffer minor or life-threatening injuries in various parts of the body. Several trauma scoring systems assess the severity of such injuries. The primary goal of this study was to assess clinical characteristics and the severity of orthopaedic, musculoskeletal, and other injuries from military training-related parachute jumping using two trauma scoring systems (AIS and ISS). Our secondary goal was to assess whether there was an increase in injury rates with age.

**Methods:**

In total, 185 military personnel between 19 and 51 years old who were injured as a result of daytime static parachute jumping during 44 months between January 2016 and August 2019 were included in the study. Demographic data; vital signs; the level of consciousness; the Glasgow Coma Scale; ISS trauma region classifications; anatomical injury sites; AIS and ISS scores; diagnoses; treatment methods; hospitalization status; and duration of hospital stay were examined descriptively.

**Results:**

There were 184 male and one female participant. The most commonly injured body site was the foot (33.5%), and the most common diagnosis was soft tissue trauma (64.3%). The most commonly injured body site was the foot (33.5%), and the most common diagnosis was soft tissue trauma (64.3%). Regarding treatment methods, 51.4% received analgesic pills and cold pack application, 42.7% received a plaster splint, and 5.9% had orthopaedic surgery. The mean ISS score was 5.16 ± 3.92. The hospitalization rate was significantly higher for patients with a critical AIS score than those with a severe AIS score *(p* < 0.001).

**Conclusions:**

The use of trauma scoring systems to assess injury severity among patients admitted to an ED due to a parachute jumping injury may facilitate treatment selection. We found that AIS and ISS were useful in determining injury severity. Therefore, we recommend the use of trauma scoring systems for assessing the injury severity and the therapeutic approach, and we advocate for the use of the 17 anatomical regions we used in this study. We also found that with increasing age, the severity of injury could increase.

## Introduction

### Background

The idea of jumping from a high place without injury first appeared in China about 2000 years ago [1]. As a result of trials conducted throughout history, the first successful parachute jump, which was a jump from a balloon, took place in 1797; subsequently, modern parachuting developed [2]. The word parachute derived from the Greek word ‘para,’ indicating protection, and the French word ‘chute,’ indicating fall [1, 2].

With the Second World War, parachuting was used and developed for military purposes. Today, many country armies still contain parachute troops. Military or hobby parachute jumps are widely used today [2, 3].

### Importance

Parachuting requires high levels of concentration and physical performance. Various injuries may occur in the air and during landing; in some cases, fatal accidents can occur. According to the literature, the rate of injuries as a result of parachute jumping is 3–24 per 1000 persons [4, 5]. The factors affecting the injury rate include altitude; whether the jump performed at night; the equipment and technique used; plane-related factors; the parachute type; the weather and other environmental factors; the ground is adequate and personal factors, such as age, weight, height, experience, aircraft departure technique and loss of control [3, 6–10]. Most parachuting accidents and fatal injuries occur at low altitudes and are due to sudden turns [3, 4, 8].

Different scoring systems used to determine the severity of trauma patients before and in the hospital. These scoring systems generally based on consciousness, vital signs, and trauma mechanisms and can be classified as physiological, anatomical, triage, or combined scoring systems. Physiological scoring systems consider consciousness and vital signs; anatomical scoring systems focus on the anatomical regions of the body, and triage scoring systems consider consciousness, vital signs, and other scoring systems. Specific scoring systems can be classified as triage scoring systems, e.g., the prehospital index (PHI) and the trauma triage rules (TTR). The anatomical scoring systems, e.g., the abbreviated injury scale (AIS), the injury severity score (ISS), the new injury severity score (NISS), the anatomic profile score (APS) and the anatomic profile. The physiological scoring systems, e.g., the revised trauma score (RTS), the Glasgow coma scale (GCS), and the acute physiology and chronic health evaluation (APACHE). Or the combined scoring systems, e.g., the trauma and injury severity score (TRISS), the organ injury scale (OIS), the international classification of diseases-based ISS (ICISS), a severity characterization of trauma (ASCOT), the trauma and injury severity score comorbidity (TRISSCOM) and the advanced trauma life support (ATLS) [11-19].

The AIS anatomical scoring system first published in 1969; it was last updated in 2015 and is still in use today. AIS can be used to reveal the epidemiological features of trauma patients. AIS also allows wound severity to compared through the use of standard terminology for the identification of wounds and simple numerical sequencing methods. According to the first AIS systems, the body anatomically divided into six regions: head (including neck), face, chest, abdomen or pelvic contents, extremities, and external. In later versions of AIS, this anatomical classification slightly expanded. AIS uses a six-point scale to classify injury severity (minor = 1, moderate = 2, serious = 3, severe = 4, critical = 5 and unsurvivable = 6) [20].

ISS is an AIS-based scoring system developed in 1974 by Baker et al. [14]. ISS provides numerical calculations and identifies the total injury severity of people with injuries to more than one body area. Like AIS, ISS divides the body into six anatomical regions, too. AIS scores for each of the three most severely injured body areas. If the ISS score is higher than 16, there is major trauma. The maximum score is 75 points, which indicates a deadly level of injury [14].

### Goal of this investigation

Due to its geographical structure, air sports, such as paragliding; military training; hobby parachuting; and balloon tours, are performed frequently in Central Anatolia and its surroundings.

In Kayseri, a city of Central Anatolia, paragliding is done for hobby and parachuting for military training. We observe an increase in the number of emergency department (ED) admissions as a result of parachute jumps has increased in recent years. In the present study, we aimed to assess the orthopaedic, musculoskeletal, and other organ-related injuries among patients admitted at the Kayseri City Training and Research Hospital Emergency Medicine Clinic, which injured as a result of jumping from parachute for military training. The primary goal of this study was to assess clinical characteristics and the severity of orthopaedic, musculoskeletal, and other injuries from military training-related parachute jumping using two trauma scoring systems (AIS and ISS) in 44 months between January 2016 and August 2019. Our secondary goal was to assess whether there was an increase in injury rates with age.

## Methods

### Study design and setting

This study was conducted at Kayseri City Training and Research Hospital, a tertiary center in Kayseri, Turkey. Ethical approval was obtained through the Kayseri City Training and Research Hospital Research Committee (16.11.2016/09) and the Erciyes University Ethics Committee (96,681,246/340). The study adhered to the Declaration of Helsinki [21].

In this descriptive study, we examined the orthopaedic, musculoskeletal, and other injuries of patients brought to the ED after parachute jumping. All patients who were injured while the emergency medicine specialist examined parachuting, and consultations were requested from relevant departments as deemed necessary. Specialists or assistant doctors performed consultations.

### Selection of participants

Total 371.324 patients admitted to our hospital ED because of all the complaints between January 2016 and August 2019. A total of 86.787 trauma patients admitted to the hospital, ED, in 2019. Of these, 200 military flight school personnel initially selected for the study. These patients were males and females between 19 and 51 years old who injured as a result of daytime static parachute jumping for military training purposes.

A total of 15 patients were excluded because they were not recorded in the hospital information management system (HIMS), their AIS and ISS trauma scores were not calculated, they did not provide consent to participate in the study and/or parts of their patient observation forms were missing. Verbal or written consent to participate in the study obtained from the remaining 185 patients.

### Measurements

For the 185 participants, demographic data, such as age and gender; ISS trauma region classification; anatomical injury sites; AIS and ISS scores; diagnoses; treatment methods; hospitalization status; and duration of hospitalization stay obtained from face-to-face interviews with the patients, HIMS and the patient registration forms. The injuries classified according to age and the trauma scoring scores. The injury rate stated as a percentage. The patients divided into three age groups: 19–23 years old (*n* = 81), 24–29 years old (*n* = 58), and 30 years old and older (*n* = 46).

We selected AIS and ISS trauma scoring since these systems allowed us to make fast and easy calculations.

### Outcomes

AIS scores calculated. ISS scores calculated as the sum of the squares of the three sites with the most severe injuries (ISS = a2 + b2 + c2); the minimum score is 1 point, and the maximum is 75 points. In the trauma regions where we thought the anatomical classification of AIS and ISS were inadequate, we divided the body into 17 different anatomical regions (head, cervical, thorax, shoulder, arm, elbow, forearm, wrist, hand, spine, abdomen, pelvic region, thigh, knee, leg, ankle, and foot) and calculated the injury rates separately.

The measurements of systolic blood pressure (SBP), which taken upon each patient’s arrival to the ED, were divided into four groups: > 100 mmHg, 86–100 mmHg, 75–84 mmHg and 0–74 mmHg. The patients’ pulse rates (P.R.s) divided into three groups: > 120/min, 51–119/min and < 50 mmHg, and their respiratory rates (R.R.s) divided into three groups: > 20/min, 10–12/min and < 10/min. The patients’ consciousness states were classified as normal, confused and meaningless speech, while the GCS scores recorded numerically.

### Data analysis

Descriptive statistics—mean ± standard deviation, median (width between quarters), and minimum-maximum for continuous variables—were used to summarize the data. Categorical variables summarized as numbers and percentages. The normality of the numerical variables checked with the Kolmogorov-Smirnov test. The Mann-Whitney U test used to compare two independent groups when the numerical variables did not show normal distribution. The Pearson chi-square test or the Fisher Freeman Halton test used to compare the categorical variables. Descriptive and frequency analyses used in the analysis of intergroup distributions. The ISS score averages compared by age group using one-way ANOVA and Spearman correlation tests.

The statistical analysis conducted by using the Jamovi version 1.0.7 software program in this study. And the significance level set at a *p*-value of 0.05 Microsoft Office Excel 2016 software used to create the graphics.

## Results

After considering exclusion criteria, a total of 185 patients included in the study, which focused on the 44 months from January 2016 to August 2019. Among the 185 participants, 99.5% (*n* = 184) were male and 0.5% (*n* = 1) were female. The mean age of the patients was 26.7 ± 6.2 years old (youngest: 19, oldest: 51). In total, 43.8% were 19–23 years old, 31.4% were 24–29 years old, and 24.8% were 30 years old and older. Among the participants, 43.7% were private soldiers, and 56.3% were ranked soldiers. It learned from the commanders that private soldiers were working on average 7 ± 2.3 months, while ranked soldiers were working on average 6 ± 1.4 years of military service. Besides, it also learned that the ranked soldiers had more parachute jumping experience.

The patients who presented to the ED with an injury complaint due to a parachute jump in 2016 (7%, *n* = 13), 2017 (39.5%, *n* = 73), 2018 (24.3%, *n* = 45) and the first 8 months of 2019 (29.2%, *n* = 54) were evaluated. The total of the patients for all conditions of ED patients was 460.716 in 2016, 464.637 in 2017, 468.223 in 2018 and 371.324 in the first 8 months of 2019. The ratio of ED patients with injury complaints due to parachute jump to other patients in 2016 was 0.003%; the ratios were 0.016% in 2017, 0.009% in 2018, and 0.015% in 2019 (Table 1).

**Table 1:** Distribution of patients by age group, gender and year

Age *Mean ± SS*	26,7 ± 6,2
**Age***n (%)*	
between ages 19–23	81 (43,8)
between ages 24–29 yaş	58 (31,4)
30 years and over	46 (24,9)
**Rank***n (%)*	
Private soldier	81 (43,7)
Rank soldier	104 (56,3)
**Gender***n (%)*	
Male	184 (99,5)
Female	1 (0,5)
**Patient application rates due to parachuting injury by years***n (%)*	
2016	13 (7)
2017	73 (39,5)
2018	45 (24,3)
2019	54 (29,2)
**Proportion of parachuting injury to all other emergency patients by years***n (%)*	
2016	13 (0,003)
2017	73 (0,016)
2018	45 (0.009)
2019	54 (0.015)

Descriptive statistics are given as number (%) for categorical variables and mean ± standard deviation for numerical variables

**Fig. 1:**
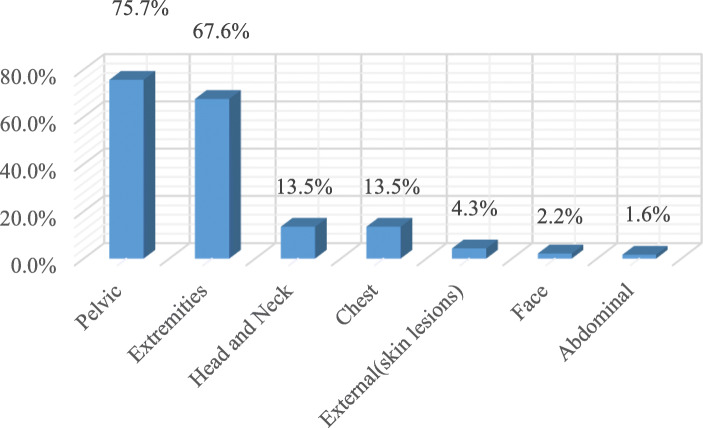
Injury rates by site, according to the ISS anatomical classifications

**Fig. 2:**
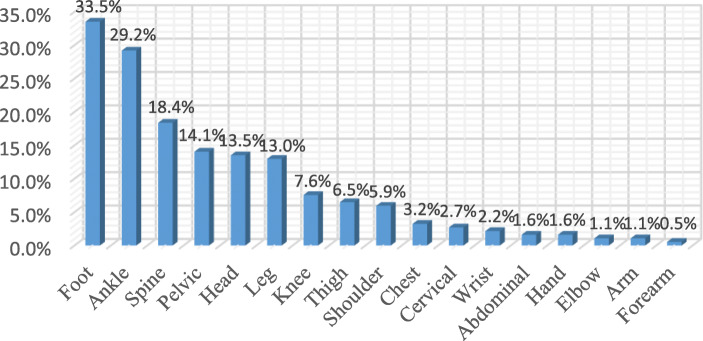
Injury rates by sites, according to the our anatomical classifications

**Fig. 3:**
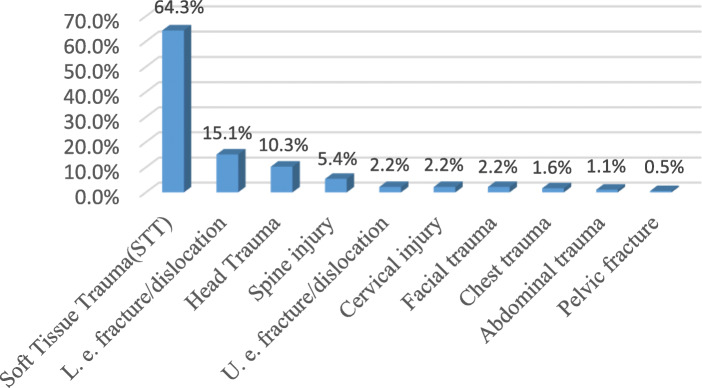
Diagnosis frequency of patients

The most common injury site was the foot (33.5%, *n* = 62), followed by the ankle (29.1%, *n* = 54), the spine (18.3%, *n* = 34) and the head (12.4%, *n* = 23). The ISS anatomical classifications of the patients’ injury sites were the abdomen/pelvis (75.7%), the extremity (67.6%), the head/neck (13.5%), and the chest (13.5%). When the injury sites were examined, 33.5% of the foot injuries were assessed as intensive, along with 29.2% of the ankle injuries, 18.4% of the spine injuries, 14.1% of the pelvis injuries, 13.5% of the head injuries, 13% of the leg injuries, 7.6% of the knee injuries, 6.5% of the thigh injuries and 5.9% of the shoulder injuries. In total, 64.3% of the injuries were diagnosed as soft tissue trauma (strain and stretching), followed by lower limb fracture-dislocation (15.1%), head trauma (10.3%), and spinal injury (5.4%). Regarding the treatment methods, 51.4% (*n* = 95) were given medication (Nonsteroidal anti-inflammatory drugs (NSAID), paracetamol pills and topical analgesic gel) and cold pack application, 42.7% (*n* = 79) received a plaster splint and 5.9% (*n* = 11) had surgery (Table 2, Figs. 1, 2 and 3).

**Table 2:** Distribution of trauma site, injury site, diagnosis and treatment method applied according to ISS

	n (%)
**Rates of injury sites by anatomical classification of the ISS**	
Pelvic, *yes*	140 (75,7)
Extremities, *yes*	125 (67,6)
Head neck, *yes*	25 (13,5)
Chest, *yes*	25 (13,5)
Subcutaneous superficial, *yes*	8 (4,3)
Face, *yes*	4 (2,2)
West, *yes*	3 (1,6)
**Injury Site**	
Foot, *yes*	62 (33,5)
Ankle, *yes*	54 (29,2)
Spine, *yes*	34 (18,4)
Pelvic region, *yes*	26 (14,1)
Head *yes*	25 (13,5)
Leg, *yes*	24 (13)
Knee, *yes*	14 (7,6)
Thigh, *yes*	12 (6,5)
Shoulder, *yes*	11 (5,9)
Thorax, *yes*	6 (3,2)
Cervical, *yes*	5 (2,7)
Wrist, *yes*	4 (2,2)
Abdomen, *yes*	3 (1,6)
Hand, *yes*	3 (1,6)
Elbow, *yes*	2 (1,1)
Arm, *yes*	2 (1,1)
Forearm, *yes*	1 (0,5)
**Diagnosis**	
STT, *yes*	119 (64,3)
Lower limb fracture, dislocation, *yes*	28 (15,1)
Head injury, *yes*	19 (10,3)
Spinal injury, *yes*	10 (5,4)
Upper limb fracture, dislocation, *yes*	4 (2,2)
Cervical injury, *yes*	4 (2,2)
Facial trauma, *yes*	4 (2,2)
Abdominal injury, *yes*	2 (1,1)
Thoracic injury, *yes*	3 (1,6)
Pelvis fracture, *yes*	1 (0,5)
**Treatment method applied**	
Medication and cold application	95 (51,4)
Splint plaster	79 (42,7)
Surgery	11 (5,9)

Descriptive statistics are given as numbers (%). *STT* Soft Tissue Trauma

Thirteen of the participants (7%) had SBPs between 75 and 84 mmHg, 144 (77.8%) had SBPs between 86 and 100 mmHg and 28 (15.1%) had SBPs > 100 mmHg. PR was > 120/min in 32 patients (17.3%) and 51–119/min in 153 patients (82.7%). RR was < 10/min in 1 patient (0.5%), > 20/min in 17 patients (9.2%) and 10–12/min in 167 patients (90.3%). Regarding level of consciousness, 1 patient (0.5%) was confused, and the others were assessed as normal. One patient (0.5%) had a GCS of 13, 10 patients (5.5%) had a GCS of 14 and 174 patients (94%) had a GCS of 15.

The mean ISS score of the patients was 5.16 ± 3.92 (minimum = 1, maximum = 25). The most frequent ISS trauma region was the extremity, followed by the pelvis (67.6%, *n* = 125), the head/neck (8.6%, *n* = 16) and the chest (7.0%, *n* = 13). Injuries were rarely observed in the abdomen (1.6%, *n* = 3).

According to the ISS anatomical classification, shoulder injuries (50%, *n* = 9) were the most commonly observed upper extremity injuries, followed by injuries to the whole upper extremity, the arm, the elbow, the hand and the wrist (the whole upper extremity *n* = 18). The most common lower extremity injuries were foot injuries (24.1%, *n* = 32), followed by ankle (13.5%, *n* = 18), leg (7.5%, *n* = 10), knee (5.3%, *n* = 7), thigh (3.8%, *n* = 5), whole lower extremity (31.6%, *n* = 42) and pelvis (14.3%, *n* = 19) injuries (Table 3).

**Table 3:** Injury rates of upper-lower extremity, head/neck, spinal and body group according to the ISS anatomical classifications

	n (%)
**ISS Upper Extremity group**	
Hand	1 (5,6)
Wrist	1 (5,6)
Elbow	2 (11,1)
Arm	2 (11,1)
Whole upper extremity	3 (16,7)
Shoulder	9 (50)
**ISS Lower Extremity group**	
Foot	32 (24,1)
Ankle	18 (13,5)
Leg	10 (7,5)
Knee	7 (5,3)
Thigh	5 (3,8)
Whole lower extremity	42 (31,6)
Pelvis	19 (14,3)
**ISS head and neck spinal group**	
Head *yes*	25 (13,5)
Neck, *yes*	2 (1,1)
Spine, *yes*	34 (18,4)
**ISS Body group**	
Thorax, *yes*	6 (66,7)
Abdomen, *yes*	3 (33,3)
Skin, subcutaneous superficial, *yes*	1 (11,1)

Descriptive statistics are given as numbers (%)

Similarly, the most common head, neck, and spinal injuries were to the spine (18.4%, *n* = 34), the head (13.5%), and the neck (1.1%). The most common injuries in the body group were thoracic (66.7%, *n* = 6), abdominal (33.3%, *n* = 3) and cutaneous-subcutaneous superficial (11.1%, *n* = 1) injuries (Table 3).

Using the AIS classifications, 22.2% (*n* = 41) of the patients’ injuries were mild, 62.2% (*n* = 115) were moderate, 15.1% (*n* = 28) were severe and 0.5% (n = 1) were critical; no deadly injuries were observed. The median ISS score was 4, and the mean was 5.16 ± 3.92 (minimum = 1, maximum = 25) (Table 4).

**Table 4:** AIS classifications and ISS scores

	*n (%)*
**AIS score**	
Mild	41 (22,2)
Moderate	115 (62,2)
Severe	28 (15,1)
Critical	1 (0,5)
**ISS score**	
*Median [IQR]*	4 [4–8]
*Min.-Max*	1–25

Descriptive statistics were given as number (%) for categorical variables and mean ± standard deviation and minimum-maximum for numerical variables

Although the average ISS scores (minimum = 1, maximum = 25) increased with age, no statistically significant difference was observed when examining these scores by age group using one-way ANOVA. There was also no statistically significant relationship found between patients’ age values and ISS values when using Spearman correlation (*p* = 0.108) (Table 5).

**Table 5:** Average ISS score by age group

*Tukey HSD*^*a,b*^		
Age groups	N	Subset for alpha = 0.05(ISS mean score)
		1
Between 19 and 23 years old	81	4‚6667
Between24–29 years old	58	5‚0862
30 years old and above	46	6‚1304
Sig.		,108

Means for groups in homogeneous subsets are displayed

^a^Uses Harmonic Mean Sample Size = 58,450

^b^The group sizes are unequal. The harmonic mean of the group sizes is used. Type I error levels are not guaranteed

Meanwhile, regarding injury sites, the rate of leg injury in patients 30 years and older were found to be statistically more significant than in patients between 24 and 29 years old (*p* = 0.043). Also, in patients between 19 and 23 years old, the rate of knee injury was statistically more significant than for those between 24 and 29 years old and those over 30 years old (*p* = 0.006). Furthermore, there were no statistical differences between the foot, ankle, thigh, pelvis, spine, abdomen, thorax, shoulder, hand, ankle, forearm, elbow, arm, cervical and head injury rates when compared by age group (*p* > 0.05 for each) (Table 6).

**Table 6:** Comparison of injury sites by age group

	Between ages 19–23	Between ages 24–29	30 and older	***p***
**Injury site**				
Foot, *yes*	31 (38,3)	21 (36,2)	10 (21,7)	0,144*
Ankle, *yes*	27 (33,3)	19 (32,8)	8 (17,4)	0,127*
Leg, *yes*	11 (13,6)a,b	3 (5,2)b	10 (21,7)a	0,043*
Knee, *yes*	12 (14,8)a	1 (1,7)b	1 (2,2)b	0,006**
Thigh, *yes*	7 (8,6)	2 (3,4)	3 (6,5)	0,504**
Pelvis, *yes*	8 (9,9)	11 (19)	7 (15,2)	0,304*
Spine, *yes*	13 (16)	14 (24,1)	7 (15,2)	0,390*
Abdomen, *yes*	2 (2,5)	1 (1,7)	0 (0)	0,789**
Thorax, *yes*	4 (4,9)	2 (3,4)	0 (0)	0,428**
Shoulder, *yes*	3 (3,7)	6 (10,3)	2 (4,3)	0,266**
Hand, *yes*	1 (1,2)	1 (1,7)	1 (2,2)	0,999**
Wrist, *yes*	1 (1,2)	1 (1,7)	2 (4,3)	0,463**
Forearm, *yes*	1 (1,2)	0 (0)	0 (0)	0,999**
Elbow, *yes*	2 (2,5)	0 (0)	0 (0)	0,500**
Arm, *yes*	0 (0)	1 (1,7)	1 (2,2)	0,318**
Cervical, *yes*	4 (4,9)	1 (1,7)	0 (0)	0,316**
Head, *yes*	8 (9,9)	6 (10,3)	11 (23,9)	0,059*

Descriptive statistics were given as numbers (%)

*. Pearson Chi-Square test was used

**. Fisher Freeman Halton test was used

There was a statistically significant difference in the hospital admission rates based on the AIS scores (*p* < 0.001); the hospitalization rate of patients with a critical AIS score was significantly higher than those with a severe AIS score. Similarly, the difference between the median ISS scores was statistically significant based on their hospitalization status (*p* < 0.001). Accordingly, the median ISS score was significantly higher among hospitalized patients than those who were not hospitalized (Table 7).

**Table 7:** Comparison of hospitalization rates based on AIS scores and ISS score medians and hospitalization duration, according to the AIS score

		Hospitalization Status		***p***
		Yes	No	
**AIS score**	Mild	0 (0)	41 (100)	< 0,001*
	Moderate	0 (0)	115 (100)	
	Severe	10 (35,7)	18 (64,3)	
	Critical	1 (100)	0 (0)	
**ISS score**		9 [9–18]	4 [4–6]	< 0,001**
		**Hospitalization days**		
**AIS score**	Mild	**–**		
	Moderate	**–**		
	Severe	3 [1–4]		
	Critical	6 [6–6]		

*. Fisher Freeman Halton test was used. Descriptive statistics are given as numbers (%)

**. Mann-Whitney U test was used. Descriptive statistics are given as median [IQR]

While 5.9% (*n* = 11) of the patients received inpatient treatment, 94.1% (*n* = 174) discharged with outpatient treatment (NSAID or paracetamol pills, topical analgesic gel, and cold pack application). Furthermore, 54.5% (*n* = 6) of the inpatients had lower extremity injuries, 27.3% (*n* = 3) had upper extremity injuries and 18.2% (*n* = 2) had shoulder surgery, which was performed by orthopaedist. Of these patients, 18.1% (*n* = 2) were hospitalized in the ICU, and 81.9% (*n* = 9) were hospitalized in the ED. None of the hospitalized patients died. The average length of hospitalization was 3.45 ± 2.20 days (minimum = 1, maximum = 7). Also, in Table 7, the median day of hospitalization of patients with a severe AIS score was 3 days, while the median day of hospitalization of critical patients was 6 days.

## Discussion

Various injuries can occur during parachute training and jumping. Due to advances in developing parachute equipment and landing techniques, injuries tend to decrease day by day [5, 7–9]. Leggat and Smith stated that musculoskeletal disorders (MSD) are a common occurrence among soldiers and represent a valuable source of morbidity for the military as a whole. Intrinsic risk factors linked to military training injuries include a diverse range of inherent variables, such as the level of prior physical conditioning, psychological makeup, age, height, weight, and gender. Extrinsic risk factors for military MSDs include training surface, exercising when fatigued, progressive training in place of cyclical training, and the type of footwear usually worn. Other military-specific variables may include drill methods, the arrangement of platoons, training techniques, and the actual training distance [22]. In our study, we think that the rate of injury as a result of jumping from a parachute varies depending on the age, experience, landing technique, and the ground descended.

The average age of the patients included in the study was 26.70 ± 6.24 years old. In Ekeland’s study, the average age was 24.5 ± 3.6 years old, and the age ranges in that study, and our study were similar [4]. The rate of injury increased with age in our study. This rate was similar to the literature but, severe and critical patient rates were higher (15.1 and 0.5%, respectively) [4, 5]. This difference may be because more people participated in our study, and the average age of the participants was higher.

The vitals of the majority of the participants in our study was generally stable, which was similar to the results found in Ekeland’s study [4].

There are differences in the frequency of age and diagnosis of injuries caused by parachuting in literature studies. In our study, the patients were most frequently diagnosed with soft tissue trauma (sprain and stretching) (64.3%, *n* = 119) and lower limb fracture-dislocation (15.1%, *n* = 28). The percentage of these diagnoses is 64.3 and 15.1%, respectively. These rates were higher than those in a study by Craig et al. [3]. The reasons for this high rate in our study may be because most soldiers (43.7%) were between the ages of 19–23 and inexperienced of the soldiers, the landing technique problems, and the inappropriate landing area selection. In our study, patients aged 30 years old and older had a statistically significantly higher leg injury rate than patients aged 24–29 years old (*p* = 0.043). In patients between 19 and 23 years old, the knee injury rate was statistically significantly higher than those between 24 and 29 years old and those over 30 years old (*p* = 0.006). These findings are similar to the literature, which we believe was due to increases in parachuting experience with age. However, proper techniques used during descent, age, and joint, bone, muscle, and ligament degeneration may also be factors in the development of leg injuries. Meanwhile, we think that the reason for the higher rate of knee injuries among younger patients may have been due to not using the five-point technique.

The rate of foot injury of the participants in our study was higher than in previous studies [4, 7, 10]. In a comprehensive study conducted by Ekeland, the most common injury site was the ankle (36%) [4]. In our study, when the foot and ankle sites were evaluated together, the injury rate reached 62.6%. When the two injuries evaluated together, similar to this study, the rate of foot and ankle injury is most common. Similarly to our study, in a retrospective study by Ball et al., which examined injuries due to static parachute jumps, the lower extremity was the most frequently injured body region [23].

Bricknell reported in his study that the injury rate increases when wind speed exceeds nine knots and during high altitude jumps [7]. In our study, we learned that from patients commanders, they each jumped with a self-opening mushroom-shaped static parachute from a plane at an altitude of approximately 400 m (1200 ft), using a five-point technique at 1–2 knots wind speed, 5.9–7.1 km/h landing speed, and they landed on flat ground. Although the wind speed was not high, we think that the injury rate was high, maybe due to improper landing techniques and the fact that most of the participants were private soldiers, and the parachute experience was low. Besides, the equipment of all of the jumpers was complete and suitable for jumping.

Static, free fall and high altitude-low opening (HALO) jumps specified by the United States (US) Army as jumping techniques used by special forces [3]. In literature studies, it was reported that 141 injuries detected during 134 HALO jumps [3, 8]. Meanwhile, in two US and British army studies, approximately 2.5% of parachute jumps resulted in injuries, the majority of which were minor [1, 8]. The injury rate for static jumps has been specified as 8.1/1.000 [3].

The patients in our study typically engaged in self-opening and low-altitude jumping. Since the total number of people who jumped was not known, the injury rate could not be calculated. However, we did determine that, during the four-year study period, and an average of 0.011% of the ED patients admitted for parachute jumping-related trauma injuries. In our study, 22.2% of the 185 patients suffered mild injuries, while 62.2% had moderate, 15.1% had severe, and 0.5% had critical injuries. Besides, 43.7% of the participants were private soldiers, and 56.3% were ranked soldiers. The ranks of those who jumped in detail in the literature studies were not fully specified, like our study.

Scoring systems, such as GCS, AIS, ISS, TRISS, and RTS, are used to assess the severity of trauma patients in EDs [12, 13, 17, 18, 20]. We chose to use AIS and ISS scores, which can be easily calculated to evaluate the severity of injuries of patients and which we think is appropriate for crowded EDs such as ours. Since we had difficulties calculating the ISS score for multiple injuries in the same region, we assessed injury severity by dividing the body into 17 different anatomical areas, noting the injury areas and evaluating them using AIS and ISS. The mean of the participants ISS was 5.16 ± 3.92 in our study. This mean was lower than the mean in a study by Sozuer et al. (8.15 ± 4.29) [24]. We think that the reason for this difference is due to the more significant number of patients included in our study and the more severe injuries of those patients.

The most commonly observed ISS anatomically trauma region was the extremity and the pelvis (67.6%, *n* = 125), followed by the head/neck (8.6%, *n* = 16) and the chest (7.0%, *n* = 13). Injuries rarely observed in the abdomen (1.6%, *n* = 3). The rates of injury site separation compared to ISS were higher in our study than in the literatüre. Compared to the ISS anatomically trauma region exposed, in our study, the most frequent was extremity and pelvis, with a rate of 67.6% (*n* = 125). Also, the least number of injuries observed in the abdomen (*n* = 3) with 1.6%. We have found that injury site separation rates are higher than in previous literature studies [3–5, 7, 24]. It may be because 43.7% of the injured are private soldiers who do not have sufficient experience.

Previous studies in the literature on parachute injuries are usually in the form of a case report or a literature summary [1, 7, 9, 25, 26]. In our study, 28 patients had lower extremity fractures, four patients had upper extremity fractures, and one patient had pelvic fractures. The fractures of the upper extremity related to parachute jumping are generally reported as a humeral surgical neck fracture or stable humerus fracture in Kirby’s study [27]. Also, in our study, the most common shoulder injury was observed in the upper extremity, and the fracture was found in 4 people. Kirby was observed that the most common foot and ankle injuries were similar to our study [27]. In one study, the ankle injury rate was calculated as 0,45% [10]; in our study, the ankle injury rate was 29.1%. This injury rate difference may be due to the larger number of patients included in our study. In one study, the ankle injury rate was calculated as 4.5/1000 [10]; in our study, the ankle injury rate was 29.1%. This injury rate difference may be due to the larger number of patients included in our study. In a retrospective study by Zakowski et al., the injury rate was 1.48–3.76/1000 jumps [26]. In our study, 11 people were operated on by orthopaedists, and 54.5% (*n* = 6) had lower extremity surgery, 27.3% (*n* = 3) had upper extremity surgery and 18.2% (*n* = 2) had shoulder surgery. Of these patients, 18.1% (*n* = 2) were hospitalized in the ICU, and 81.9% (*n* = 9) were hospitalized in the ED.

In a study by Farrow, 63 of 8886 jumps resulted in death, and the injury rate was 7.1/1000 [5]. In our study, there were no fatal cases. Westman et al. examined deadly events due to free fall building-antenna-span-earth (BASE) jumping between 1981 and 2006 and found that the deaths were related to human, environment and equipment problems, reverse airflow, loss of vision due to weather and acrobatic movements [6]. Another study found that similar factors affected the injury rate and also found a high injury rate for night-time jumps [9]. Since the patients in our study did not perform night-time jumps, the injury rate of night-time jumps could not be calculated. In the literature, an incorrect landing technique was mentioned as the most common cause of injury [4, 23]. Since the night jump was not performed in our study, the rate of injury in the night jump could not be calculated. It is pleasing that no fatal case was encountered in our study. It may be because the soldiers did not prefer to jump out of the parachute while the weather was windy and badly, and a lower altitude was preferred for training. We also think that all of the jumping during the daytime could be an effective factor on the absence of fatal injury.

According to the literature, hospitalization and mortality are associated with increases in AIS and especially ISS scores [14, 16, 28, 29]. In our study, as the AIS and ISS scores increased, the rate of hospitalization increased. Therefore, the use of trauma scoring systems can be interpreted as facilitating the work of emergency staff in determining the severity of trauma, hospitalization decisions, and possible mortality risks.

In the literature, it has been stated that musculoskeletal disorders (MSD) are a common occurrence for soldiers and an essential source of morbidity for the army as a whole [22]. Regarding the average age of the participants in our study, 43.8% were 19–23 years old, 31.4% were 24–29 years old, and 24.8% were 30 years old and older. Although there was an increase in ISS score with age in our study, there was no statistically significant difference. We think that the reason for this is that, as age increases, attention may decrease due to an increase in self-confidence as the person gains experience, which, in turn, may cause an increase in injury rates.

### Strengths and limitations

Our study has some limitations. Since we wanted to evaluate and compare cases of isolated parachute injuries, our study did not include a control group. We did not consider the jumpers how many times they had jumped before. And, how many soldiers had been injured on the same day were not questioned. We learned from the soldiers’ commander that all of the jumps occurred in the daytime. Since only soldiers who fall from the parachute during the day are included in our study, we do not know the injury characteristics that occur as a result of parachuting at night. In later studies, day and night parachute jumps can be compared.

After the emergency treatments, only hospitalized patients continued to be followed, and outpatients did not follow after they discharged from the ED For this reason, it did not know whether any of the patients had any complications after discharge. Besides, only parachute and related injuries were included in the study; paragliding and balloon accidents did not included. Since all jumps were made in the form of a static line, injury rates due to free jumps were not studied.

In terms of the strengths of the study, we conducted this prospective study over a 44-week observation period, included many cases, detailed the anatomical locations of the parachute injuries, used two different scoring systems to assess the severity of the trauma, collected data on treatment measures and recorded the patients’ hospital stay duration. Also, as the scoring systems were considered insufficient, we used more detailed classifications of the injury sites.

## Conclusions

Various injuries can occur during parachute training and jumping. Due to advances in developing parachute equipment and landing techniques, injuries tend to decrease day by day. We believe that parachute jumping-related injury rates will be reduced by increasing the first aid training given to parachute instructors and students and by further improving parachute jumping techniques. However, although new jumping techniques and equipment developed, we think that the most critical factors on the parachute-related injury are the human factors.

On the other hand, emergency workers should be kept in mind that injuries due to air sports are more common in inexperienced people and that more severe injuries may occur in people with advanced age. Therefore, we suggest that military personnel over the age of 30 behave more cautiously when jumping from a parachute and especially during a landing. We also believe that injury rates may decreased by correcting risk factors linked with the person and the environment.

As a result, parachuting and air sports are becoming more popular day by day. For the asses injury severity of the trauma caused by these jumps made for military or sports purposes and the planning of the treatment, we recommend that the emergency departments use a classification of trauma scores systems alone, combined, or divide the body into more anatomical regions as we use.

## Data Availability

The datasets analyzed during the current study are available from the corresponding author on reasonable request.

## Abbreviations

AIS: Abbreviated Injury Scale
APS: Anatomic Profile Score
ASCOT: A Severity Characterization of Trauma
GCS: Glasgow Coma Score
HALO: High Altitude-Low Opening
HBYS: Hospital Information Management System
ICU: Intensive Care Unit
ISS: Injury Severity Score
NSAID: Nonsteroidal anti-inflammatory drugs
NISS: New Injury Severity Score
OIS: Organ Injury Scale (OIS),
PR: Pulse Rate
RR: Respiratory Rate
RTS: Revised- Trauma Score
SBP: Systolic Blood Pressure
STT: Soft Tissue Trauma
TRISS: Trauma and Injury Severity Score
TRISSCOM: Trauma and Injury Severity Score Comorbidity

## References

[1] Davison DJ (1990). A review of parachuting injuries. Injury..

[2] Lucas J (1997). The silken canopy: a history of the parachute.

[3] Craig SC, Lee T (2000). Attention to detail: injuries at altitude among U.S. Army Military Static Line Parachutists. Mil Med.

[4] Ekeland A (1997). Injuries in military parachuting: a prospective study of 4499 jumps. Injury..

[5] Farrow GB (1992). Military static line parachute injuries. Aust N Z J Surg.

[6] Westman A, Rosén M, Berggren P, Björnstig U (2008). Parachuting from fixed objects: descriptive study of 106 fatal events in BASE jumping 1981–2006. Br J Sports Med.

[7] Bricknell MCM, Craig SC (1999). Military parachuting injuries: a literature review. Occup Med (Chic Ill).

[8] Glorioso JE, Batts KB, Ward WS (1999). Military free fall training injuries. Mil Med.

[9] Knapik J, Steelman R (2016). Risk factors for injuries during military static-line airborne operations: a systematic review and meta-analysis. J Athl Train.

[10] Schumacher JTJ, Creedon JF, Pope RW (2000). The effectiveness of the parachutist ankle brace in reducing ankle injuries in an airborne ranger battalion. Mil Med.

[11] Wisner DH (1992). History and current status of trauma scoring systems. Arch Surg.

[12] Maslanka AM (1993). Scoring systems and triage from the field. Emerg Med Clin North Am.

[13] Senkowski CK, McKenney MG (1999). Trauma scoring systems: a review. J Am Coll Surg.

[14] Baker SP, O’Neill B, Haddon WJ, Long WB (1974). The injury severity score: a method for describing patients with multiple injuries and evaluating emergency care. J Trauma.

[15] Galvagno SM Jr, Massey M, Bouzat P, Vesselinov R, Levy MJ, Millin MG, et al. Correlation Between the Revised Trauma Score and Injury Severity Score: Implications for Prehospital Trauma Triage. Prehospital emergency care: official journal of the National Association of EMS Physicians and the National Association of State EMS Directors. 2019;23(2):263–270. 10.1080/10903127.2018.1489019.

[16] Roy N, Gerdin M, Schneider E, Veetil DKK, Khajanchi M, Kumar V (2016). Validation of international trauma scoring systems in urban trauma centres in India. Injury..

[17] Greenspan L, McLELLAN BA, Greig H (1985). Abbreviated injury scale and injury severity score: a scoring chart. J Trauma.

[18] Baker SP, O’neill B (1976). The injury severity score: an update. J Trauma Acute Care Surg.

[19] Copes WS, Champion HR, Sacco WJ, Lawnick MM, Gann DS, Gennarelli T (1990). Progress in characterizing anatomic injury. J Trauma.

[20] Loftis KL, Price J, Gillich PJ (2018). Evolution of the abbreviated injury scale: 1990–2015. Traffic Inj Prev.

[21] Williams JR (2008). The declaration of Helsinki and public health. Bull World Health Organ.

[22] Leggat PA, Smith DR (2007). Military training and musculoskeletal disorders. J Musculoskelet Pain.

[23] Ball VL, Sutton JA, Hull A, Sinnott BA (2014). Traumatic injury patterns associated with static line parachuting. Wilderness Environ Med.

[24] Sozuer EM, Ozkan S, Akdur O, Durukan P, Ikizceli I, Avsarogullari L (2008). Injuries due to parachute jumping. Ulus Travma Acil Cerrahi Derg.

[25] Bourghli A, Fabre A (2017). Proximal end clavicle fracture from a parachute jumping injury. Orthop Traumatol Surg Res.

[26] Zakowski B, Wagner I, Domzalski M (2019). Analysis of a military parachutist injury–a retrospective review of over 37,000 landings. Mil Med.

[27] Kirby CN. Parachuting injuries. Proc R Soc Med. 1974;67(1):17–21. 10.1177/003591577406700112 PMC1645705 20919151

[28] Narcı A, Solak O, Turhan-Haktanır N, Ayçiçek A, Demir Y, Ela Y (2009). The prognostic importance of trauma scoring systems in pediatric patients. Pediatr Surg Int.

[29] Mullins RJ, Veum-Stone J, Helfand M, Zimmer-Gembeck M, Hedges JR, Southard PA (1994). Outcome of hospitalized injured patients after institution of a trauma system in an urban area. Jama..

